# Coxsackievirus-B4 Infection of Human Primary Pancreatic Ductal Cell Cultures Results in Impairment of Differentiation into Insulin-Producing Cells

**DOI:** 10.3390/v11070597

**Published:** 2019-07-02

**Authors:** Antoine Bertin, Famara Sane, Valery Gmyr, Delphine Lobert, Arthur Dechaumes, Julie Kerr-Conte, François Pattou, Didier Hober

**Affiliations:** 1 University of Lille, Faculté de Médecine, CHU Lille, Laboratoire de Virologie EA3610, F-59000 Lille, France; 2 University of Lille, INSERM, CHU Lille, EGID, UMR 1190- Translational Research in Diabetes, F-59000 Lille, France

**Keywords:** enterovirus, in vitro, c-peptide, insulin mRNA, RT-PCR

## Abstract

Coxsackievirus-B4 (CV-B4) E2 can persist in the pancreatic ductal-like cells (Panc-1 cell line), which results in an impaired differentiation of these cells into islet-like cell aggregates (ICA). In this study, primary pancreatic ductal cells obtained as a by-product of islet isolation from the pancreas of seven brain-dead adults were inoculated with CV-B4 E2, followed-up for 29 days, and the impact was investigated. Viral titers in culture supernatants were analyzed throughout the culture. Intracellular viral RNA was detected by RT-PCR. Levels of ductal cell marker CK19 mRNA and of *insulin* mRNA were evaluated by qRT-PCR. The concentration of c-peptide in supernatants was determined by ELISA. Ductal cells exposed to trypsin and serum-free medium formed ICA and resulted in an increased insulin secretion. Ductal cells from five brain-dead donors were severely damaged by CV-B4 E2, whereas the virus persisted in cultures of cells obtained from the other two. The ICAs whose formation was induced on day 14 post-inoculation were scarce and appeared tiny in infected cultures. Also, *insulin* mRNA expression and c-peptide levels were strongly reduced compared to the controls. In conclusion, CV-B4 E2 lysed human primary pancreatic ductal cells or persisted in these cells, which resulted in the impairment of differentiation into insulin-producing cells.

## 1. Introduction

Type 1 diabetes (T1D), a multifactorial disease characterized by a defective production of insulin by pancreatic beta cells, occurs in genetically predisposed subjects [1]. Enteroviruses, especially coxsackieviruses-B (CV-B), are associated with T1D [2]. This has been proved by the presence of enteroviral RNA and/or protein in blood, intestine, and/or pancreas of patients with T1D [3]. Nevertheless, the role of these viruses in the pathogenesis of the disease and the underlying mechanisms have not been deciphered yet [4]. Persistent CV-B infection and impaired beta cells regeneration are some of the mechanisms possibly involved [3,5,6,7]. Interestingly, enteroviral RNA has been detected in pancreatic ductal cells of patients with T1D [8]. The CV-B4 E2 strain used was isolated from the pancreas of a patient who died shortly after the onset of the disease, and this virus caused T1D in mice [9].

Insulin producing cells have been observed in pancreatic ducts, which hypothesizes beta cell neogenesis from pancreatic ductal cells [10,11,12]. Previously, it was reported that ductal-like cells (Panc-1 cell line) can be trans-differentiated into islet-like cell aggregates (ICA) [13,14], and a persistent CV-B4 E2 infection of these cells will inhibit their differentiation into islet-like aggregates (ICAs) [15]. It also has been reported that CV-B4 E2 can infect in vitro human primary pancreatic ductal cells [15]. However, whether CV-B can disturb the differentiation of human primary pancreatic ductal cells or not remains an open issue. In the present study, human primary pancreatic ductal cells were infected with CV-B4 E2 and the subsequent impact was investigated.

## 2. Materials and Methods

### 2.1. Human Pancreatic Ductal Cells

In agreement with French law and the Ethical Committee of our institution, human primary ductal cells, harvested from the pancreas of seven brain-dead adults, were obtained as a by-product of human islet isolation. The automated method of Ricordi [16] with collagenase solution was applied, as previously described [15]. Briefly, after being washed in Hank’s solution, the pellet was placed in a 75 cm² dish with DMEM containing 3 g/L glucose and, supplemented with 10% fetal calf serum (FCS), and 1% insulin transferrin selenium (ITS, Sigma-Aldrich, Saint-Quentin-Fallavier, France). Geneticin (G418, Sigma-Aldrich) was used for fibroblast overgrowth limitation. After three weeks of culture, the cells were de/trans-differentiated into cytokeratin 19-positive cells [15,17]. To induce the formation of ICAs, the medium of ductal cell cultures was removed and the cell layers were washed once. Then the cultures were incubated in the presence of 0.05% trypsin for two minutes. This was followed by the removal of trypsin, the addition of serum free medium Dulbecco’s modified Eagle Medium (DMEM) F12 complemented with 1% bovine serum albumin (BSA) and the culture was pursued. When ICAs were formed (24 to 48 h later), they were harvested and cultured in 24-wells tissue culture insert plates. UptiBlue (Uptima Interchim, Montluçon, France) and trypan blue (Sigma) were used as routinely described for studying metabolic activity and cell viability, respectively.

### 2.2. Virus

The diabetogenic strain CV-B4 E2, provided by Ji-Won Yoon (Julia McFarlane Diabetes Research Center, Calgary, Alberta, Canada), was initially propagated in HEp-2 cells [15]. Viral particles were released from the cells by three freeze–thaw cycles and supernatants were collected after centrifugation at 2000*g* for 10 min at 4 °C. Aliquots of virus preparations were stored at −80 °C and virus titer was determined on the HEp-2 cells by end-point dilution assay using the Reed–Muench method.

### 2.3. Cell Infection

Human primary ductal cells were seeded in 24-well plates and infected with 2.10^3^ TCID_50_ (Median Tissue Culture Infective Dose) CV-B4 E2 on day 21 post-isolation. Plates were incubated at 37 °C in a humidified atmosphere with 5% CO_2_ for 3 h then the cell layers were washed three times with cold culture medium. After washing, ductal cell cultures were maintained in fresh DMEM at 37 °C in an incubator and supernatant samples were harvested 3 h post-inoculation (i.e., day one p.i.).

Human primary ductal cell cultures were followed-up for 29 days post-inoculation, with media changed every three days and supernatant fluids were harvested and stored at −80 °C for infectivity tests. All along the follow-up, mock and CV-B4 infected cells were collected in lysis buffer for RNA isolation.

### 2.4. RNA Isolation and Reverse Transcription-Polymerase Chain Reaction (RT-PCR)

RNA was isolated from cells to characterize ductal cells infection and gene expression using Qiagen RNeasy Mini extraction kit according to the manufacturer’s instructions. Quant-it® RNA Assay kit (Invitrogen, Illkirch, France) was used for total RNA titration on 96-well plates using the Mx3000P® system (Stratagen, Massy, France). The PCR tests were carried out in a Mastercycler® gradient (Eppendorf, Montesson, France). The Superscript ™ One-step RT-PCR with Platinum® Taq (Invitrogen), which allows one step cDNA synthesis and amplification, was used for RT-PCR, according to the manufacturer’s instructions. Amplified products were revealed by "molecular biology grade” (Eurogentec, Angers, France) agarose electrophoresis.

### 2.5. Quantitative RT-PCR

Real-time quantitative RT-PCR (RT-qPCR) was performed using the LightCycler RNA Master SYBR Green kit and the LightCycler 480 (Bio-Rad, Steenvoorde, France) to measure the mRNA expression levels of *insulin* and housekeeping genes in persistently infected ductal cells. Superscript II (Life Technologies, Illkirch, France) and Sso Advanced SYBR Supermix (Bio-Rad) were used, respectively, for reverse PCR and quantitative PCR tests as described by the manufacturer’ instructions. PCR primer sequences are listed in Table 1. All primer sets were tested to ensure that only a single product of the correct size was amplified. Water was used as negative control and run with all reactions. Relative expression was calculated after normalization with large ribosomal protein (*RPLPO*) gene which was used as an endogenous control (housekeeping gene). Cytokeratin 19 (*CK19*) is a marker of pancreatic ductal cell [16].

### 2.6. C-Peptide Quantification

The level of c-peptide in culture supernatants was determined by using C-peptide ELISA (Mercodia ®, Uppsala, Sweden) according to the manufacturer’s instructions.

## 3. Results

### 3.1. Human Primary Pancreatic Ductal Cells Can Be Differentiated into Insulin-Producing Islet-Like Cell Aggregates

**Figure 1:**
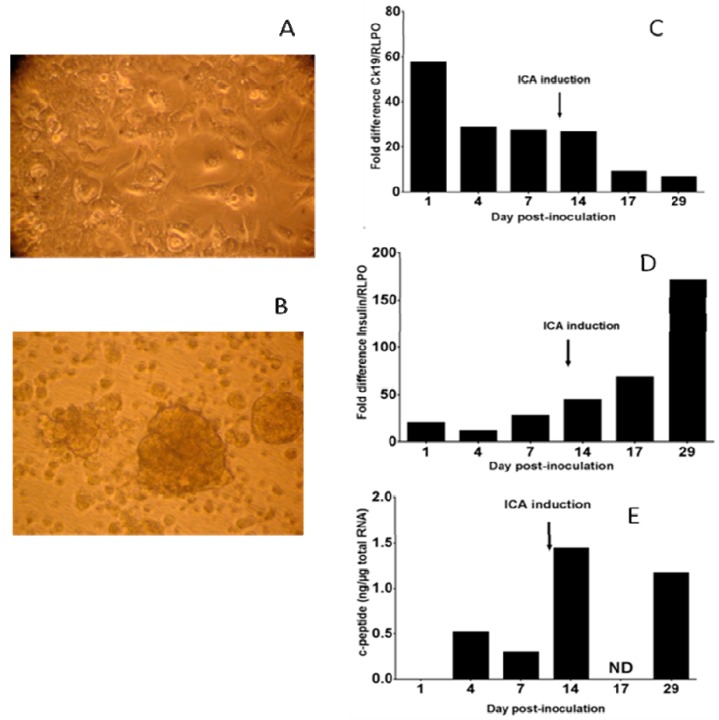
Human primary pancreatic ductal cells. Ductal cell cultures (**A**) were exposed to trypsin and serum-free medium to induce the formation of islets-like cell aggregates (ICAs) (**B**) (magnification: 40×). The levels of *CK19* and *insulin* mRNA were monitored by quantitative PCR throughout the culture (**C** and **D**). The levels of c-peptide in supernatants were determined by ELISA, the results were expressed as ng of c-peptide per µg of total RNA (**E**) (ND: not done). The representative results of two independent experiments are shown.

Human primary ductal cells were cultured for seven weeks. Cells were treated with trypsin, and medium was changed to induce ICA formation as described in the materials and methods section (Figure 1A,B). The expression of the ductal cell marker *CK19* was measured by real-time PCR, relatively to RLPO mRNA expression. After ICA formation, there was a drop in the expression of *CK19* mRNA compared to the controls (Figure 1C). In contrast, there was a rise in *insulin* mRNA normalized with housekeeping gene *RLPO* and in c-peptide in supernatants (Figure 1D,E).

### 3.2. CV-B4 E2 Can Persist in Human Primary Pancreatic Ductal Cells In Vitro

**Figure 2:**
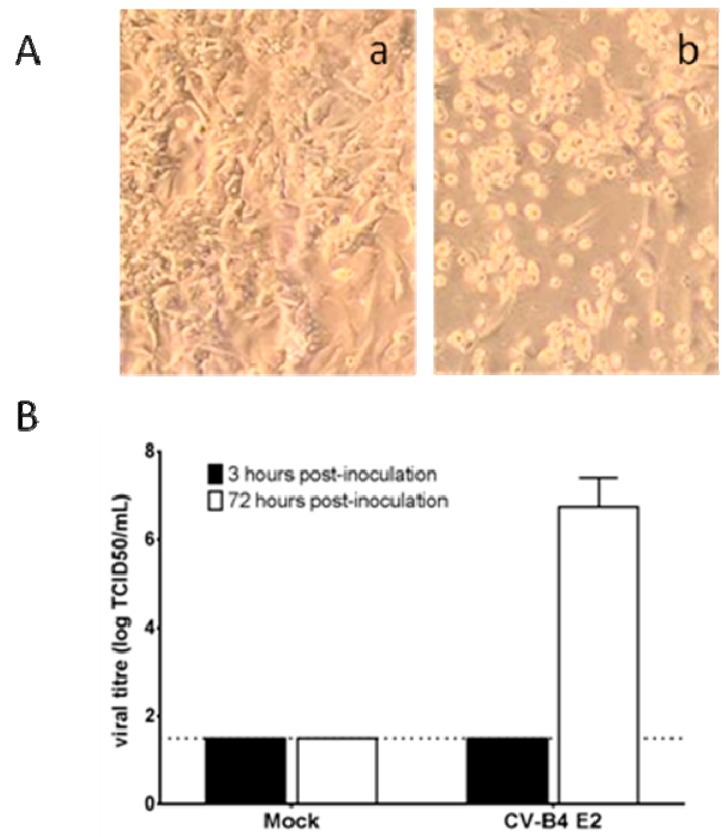
Cultures of human primary pancreatic ductal cells inoculated with CV-B4 E2. Ductal cell cultures were mock-infected or infected with CV-B4 E2. (**A**) The cell layer was observed under an inverted microscope 72 h post-inoculation: (a) mock-infected (b) infected with CV-B4 E2 (magnification: 40×). (**B**) The viral titers in culture supernatants harvested 3 h and 72 h post-inoculation were determined by plaque infectivity assay using HEp-2 cells. Results are mean + SD of five independent experiments. The limit of detection of the assay is represented by a dashed line.

Ductal cells were obtained from seven brain-dead donors. When cultures of ductal cells from five brain-dead donors were inoculated with CV-B4 E2, the cell layers were severely damaged three days post-inoculation compared to the control (Figure 2A). In these cultures, the values of viral titers ranged between 10^6.5^ and 10^7.5^ TCID50.mL^−1^ (Figure 2B). 

**Figure 3:**
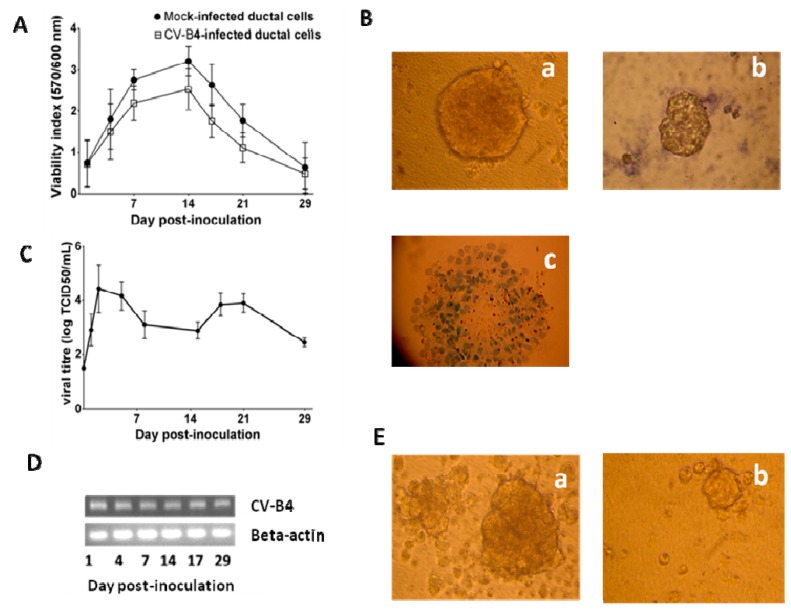
CV-B4 E2 can persist in human primary pancreatic ductal cells. Ductal cell cultures were mock-infected or infected with CV-B4 E2, followed by transformation on day 14 post-inoculation. (**A**) The viability index was evaluated using UptiBlue assay. The mean +/- SD of two experiments are shown. (**B**) The cell viability was assessed 29 days p.i. using the trypan blue exclusion assay: (a) mock-infected culture (b) CV-B4 E2-infected culture (c) mock-infected culture maintained in trypan blue for 1 hour (positive control for staining of dead cells). (**C**) The viral titers in culture supernatants were determined by plaque infectivity assay using HEp-2 cells. The results expressed as TCID50·mL^−1^, were found by titration and are the mean +/- SD values of two experiments. (**D**) Agarose gel electrophoresis of amplicons specific to the CV-B4 E2 RNA. Strand-specific RT-PCR was carried out on total RNA taken from CV-B4 E2-infected cultures at various days post-inoculation (top). The expression of *beta-actin* mRNA in each sample was investigated (bottom). (**E**) In cultures differentiated into ICAs the morphology of ICAs was evaluated 24 h after ICA induction: (a) mock-infected cultures (b) CV-B4 E2-infected cultures (magnification: 40×). B, D, and E: representative results of two experiments are shown.

By contrast, when cultures of ductal cells from two other brain-dead donors were inoculated with CV-B4 E2, the cell layers were not damaged. The metabolic activity of both mock and infected cultures, assessed by UptiBlue assay, increased up to day 14 post-inoculation. Then the formation of ICA was induced and it was observed that the metabolic activity decreased (Figure 3A). Nevertheless, on day 29 p.i. the cells were alive when analyzed with trypan blue exclusion test (Figure 3B). Infectious particles were detected in the supernatants of infected cultures up to 29 days p.i. (end of the follow-up), and the values of viral titers ranged between 10^3^ and 10^5^ TCID50.mL^−1^ (Figure 3C). The cells harbored CV-B4 E2 RNA (Figure 3D). No viral RNA was found in the ductal cells prior to the infection or in mock-infected ductal cells throughout the experiment (data not shown). The ICA structures whose formation was induced on day 14 p.i. appeared smaller in CV-B4 E2-infected cultures than in mock-infected cultures (Figure 3E). Infected and uninfected ICAs were viable, as assessed by trypan blue.

### 3.3. CV-B4 E2 Can Inhibit the Production of Insulin by Differentiated Human Primary Pancreatic Ductal Cells 

**Figure 4:**
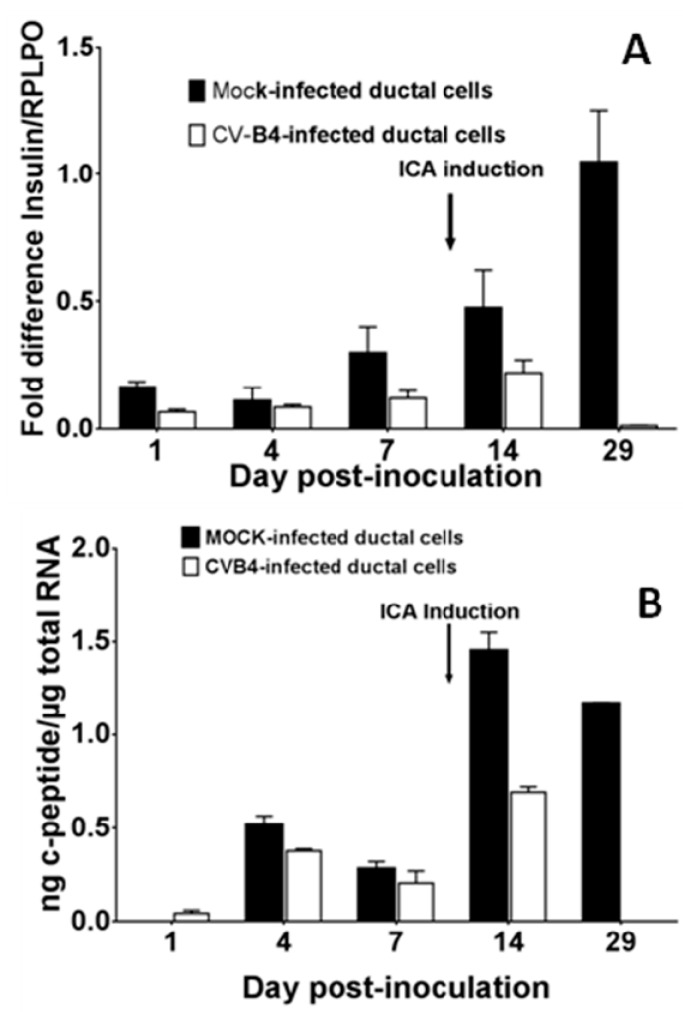
CV-B4 E2 can inhibit the production of insulin by human primary pancreatic ductal cells. Ductal cell cultures were mock-infected or infected with CV-B4 E2, and followed by transformation on day 14 post-inoculation. (**A**) *insulin* mRNA expression was quantified by RT-qPCR and normalized with *RLPO* expression. (**B**) C-peptide levels in supernatants were assessed by ELISA, and normalized with total RNA harvested from cells. Results are mean +/- SD of two independent experiments.

No difference was observed in *insulin* mRNA expression between the mock and the CV-B4 E2-infected cells prior to ICA formation in cultures of human primary ductal cells without obvious cytolysis after virus inoculation. There was no difference between the pattern of culture and differentiation of ductal cells from both donors. When ICA formation was induced, *insulin* mRNA expression was 2-fold and 100-fold higher in mock-infected cells than in CV-B4 E2-infected cells on day 14 p.i. and on day 29 p.i. respectively (Figure 4A). The levels of c-peptide in culture supernatants, measured on day 14 and 29 p.i. by ELISA and normalized with total RNA harvested from cells, were much lower in CV-B4 E2-infected cultures than in controls, which is in agreement with the inhibition of *insulin* mRNA observed in infected cultures on day 14 and 29 p.i. (Figure 4B). 

## 4. Discussion

In this study human primary pancreatic ductal cells were cultured for seven weeks. In this system, the formation of ICA can be induced and the cells can be differentiated into insulin-producing cells. The cultures were treated as described in our previous study that aimed to induce the differentiation of Panc-1 cells (human pancreas ductal cell line) [15]. The method based on the one reported by Hardikar et al. is a simple procedure, which induces stress in cells (treated with 0.05% trypsin and then cultured in serum-free medium), that is suitable for in vitro studies. In other studies, human primary ductal cells were differentiated into insulin-producing cells by treatment with bone morphogenetic protein 7 (*BMP7*), or by virus-mediated transfection to express transcription factors such as *PDX1, Neurog3,* or *Pax6* [18,19]. Other teams obtained a differentiation of human primary pancreatic ductal cells pointed out by the formation of ICAs, progressively increasing in size, three to nine days post-induction, with insulin secretion highlighted as soon as five days post-induction [18]. The differentiation of human primary pancreatic ductal cells in our experiments was highlighted by the rapid formation of ICAs observed as soon as 18 h post-induction, followed promptly by insulin secretion as evidenced by the levels of c-peptide in culture supernatants. Due to the presence of insulin in the culture medium of ductal cells, c-peptide, a by-product of insulin was measured in cultures supernatants. Moreover, the synthesis of insulin by differentiated cells was shown by the raised levels of intracellular *insulin* mRNA as compared to controls. Taken altogether the levels of intracellular *insulin* mRNA and of c-peptide in supernatants demonstrate that primary pancreas ductal cells can differentiate into insulin-producing cells in vitro in our experiments.

Cultures of human primary pancreatic duct cells were inoculated with CV-B4 E2. Every culture inoculated with the virus was productively infected as evidenced by the high viral titer in the culture supernatant. In five out of seven cases, the inoculation of CV-B4 E2 into cultures resulted in an extensive damage of cell layers three days post-inoculation, whereas mock-infected cultures were unaltered. However, the impact of CV-B4 E2 in cultures obtained from two out of seven brain-dead donors was not as dramatic. Indeed, there was no virus-induced damage of cell layer in these cases, which is in agreement with previous observations reported by our team [15]. Taken altogether our current and previous experiments indicate that there is an inter-individual variation of the effect of CV-B4 E2 in primary ductal cell cultures. This is reminiscent of results previously reported showing that the degree of islet disintegration (graded from 0 to 4) by CV-B4 E2 and other CV-B4 strains varied between different islet donors [20]. 

In previous studies, human primary pancreatic ductal cell cultures were maintained for one week whereas they were maintained for a longer period of time (up to seven weeks) in the present one [15]. CV-B4 E2 persisted in these cultures up to the end of the follow-up (four weeks p.i.). In these cultures that were maintained for several weeks and that were not damaged by CV-B4 E2, the impact of the virus on the differentiation and the production of insulin was investigated. It is noteworthy that an incomplete differentiation resulting in fewer and smaller ICAs was observed in these cultures. Moreover, in CV-B4 E2-infected cell cultures the levels of *insulin* mRNA and c-peptide were much lower compared to controls. 

The number of donors was limited, but the results indicated that CV-B4 can either damage human primary pancreatic ductal cells or can persist in these cells which results in impairment of their differentiation into insulin producing cells. Studies based on larger cohorts are needed to address this issue further and to determine which donor-related factors are involved in the impact of the virus in the pancreatic ductal cells. Whatever these factors and the pattern of infection are, which is either lytic or persistent, CV-B4 E2 can prevent the differentiation of human pancreatic ductal cells into insulin-producing cells. The system described in this study is a unique and new model that opens ways to investigate the interactions between CV-B4 E2 and human pancreatic cells, which may be involved in the pathogenesis of type 1 diabetes. Further studies will be directed along this line in our laboratory.

## Figures and Tables

**Table 1:** Primer sequences.

	Target		Sequence
RT-PCR	Beta-actin	forward	5’-GGCACTCTTCCAGCCTTCCT-3’
		reverse	5’-GCAATGCCAGGGTACATGGT-3’
	CV-B4 E2	forward	5’-CAAGCACTTCTGTTTCCCCGG-3’
		reverse	5’-ATTGTCACCATAAGCAGCCA-3’
RT-QPCR	RLPO	forward	5’-ACCTCCTTTTTCCAGGCTTT-3’
		reverse	5’-CCCACTTTGTCTCCAGTCTTG-3’
	Insulin	forward	5’-TGCATCAGAAGAGGCCATCA-3’
		reverse	5’-CGTTCCCCGCACACTAGGTA-3’
	CK19	forward	5’-CCAGCCGGACTGAAGAATTG-3’
		reverse	5’-TGGGCTTCAATACCGCTGAT-3’
